# Quinolizidine alkaloid biosynthesis: recent advances and future prospects

**DOI:** 10.3389/fpls.2012.00239

**Published:** 2012-10-26

**Authors:** Somnuk Bunsupa, Mami Yamazaki, Kazuki Saito

**Affiliations:** ^1^Graduate School of Pharmaceutical Sciences, Chiba UniversityChiba, Japan; ^2^RIKEN Plant Science CenterYokohama, Japan

**Keywords:** *Lupinus*, lysine decarboxylase, lysine-derived alkaloids, *o*-tigloyltransferase, quinolizidine alkaloids

## Abstract

Lys-derived alkaloids, including piperidine, quinolizidine, indolizidine, and lycopodium alkaloids, are widely distributed throughout the plant kingdom. Several of these alkaloids have beneficial properties for humans and have been used in medicine. However, the molecular mechanisms underlying the biosynthesis of these alkaloids are not well understood. In the present article, we discuss recent advances in our understanding of Lys-derived alkaloids, especially the biochemistry, molecular biology, and biotechnology of quinolizidine alkaloid (QA) biosynthesis. We have also highlighted Lys decarboxylase (LDC), the enzyme that catalyzes the first committed step of QA biosynthesis and answers a longstanding question about the molecular entity of LDC activity in plants. Further prospects using current advanced technologies, such as next-generation sequencing, in medicinal plants have also been discussed.

## INTRODUCTION

Plant secondary metabolites play multiple roles in the interaction between plants and their environment (Dixon, 2001). Almost 100,000 secondary metabolites have been discovered from plant species (Verpoorte, 1998; Hostettmann and Terreaux, 2000; Afendi et al., 2012). Alkaloids are organic nitrogenous bases, usually in a heterocyclic ring, with characteristic toxicity and marked pharmacological effects in humans and animals (De Luca and St Pierre, 2000). Alkaloids are derived from the products of primary metabolism, with amino acids, such as Phe, Tyr, Trp, Orn, and Lys, serving as their main precursors (Facchini, 2001). Approximately 20% of plant species accumulate alkaloids and over 12,000 different alkaloids have been described, suggesting higher structural and biosynthetic diversity compared to other secondary metabolites (Croteau et al., 2000; De Luca and St Pierre, 2000).

**FIGURE 1:**
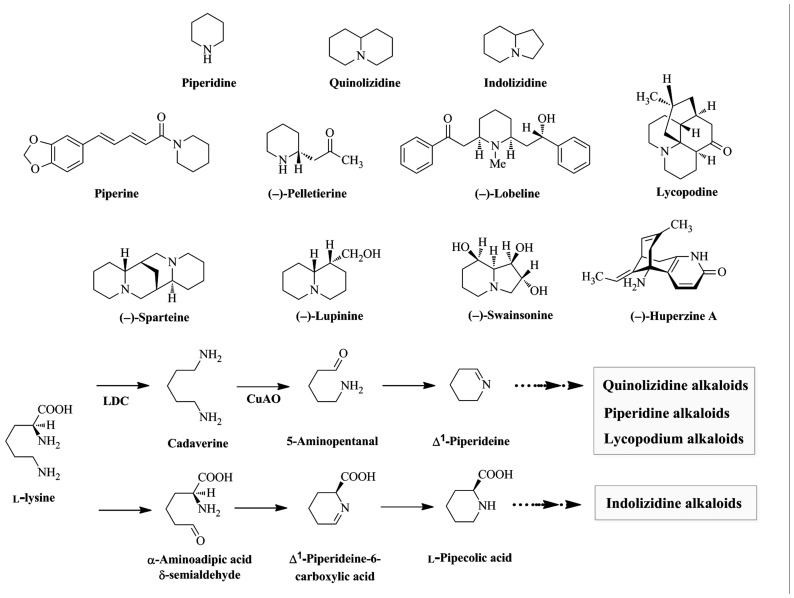
**Biosynthesis of universal intermediates, skeleton structures, and some Lys-derived alkaloids found in plants**. Piperine, (–)-pelletierine, and (–)-lobeline are piperidine alkaloids. (–)-Lupinine and (–)-sparteine are quinolizidine alkaloids. (–)-Swainsonine is an indolizine alkaloids. Lycopodine and (–)-huperzine A are lycopodium alkaloids. LDC, Lys decarboxylase; CuAO, copper amine oxidase.

Alkaloids derived from Lys are widely distributed throughout the plant kingdom and are subdivided into piperidine, quinolizidine, indolizidine, and lycopodium alkaloids. Piperidine alkaloids are found in plants in the order Piperales (e.g., piperine from *Piper nigrum*), Myrtales (e.g., (–)-pelletierine from *Punica granatum*), and Asterales (e.g., (–)-lobeline from *Lobelia inflata*). Quinolizidine (e.g., (–)-lupinine and (–)-sparteine from *Lupinus luteus* and *Cytisus scoparius*, respectively) and indolizidine (e.g., (–)-swainsonine from *Swainsona canescens*) alkaloids are found mainly in the order *Fabales* (Murakoshi et al., 1975, 1986; Colegate et al., 1979; Su and Horvat, 1981; Neuhofer et al., 1993; Felpin and Lebreton, 2004). Lycopodium alkaloids (e.g., (–)-huperzine A and lycopodine from *Huperzia serrata*) are found mainly in the genus *Lycopodium* (**Figure 1**; Liu et al., 1986; Ma and Gang, 2004). Several alkaloids have beneficial properties for humans and are used in medicine. For example, (–)-lobeline is used in the treatment of central nervous system disorders and drug abuse and (–)-huperzine A is used in the treatment of Alzheimer’s disease (Decker et al., 1992; Bai et al., 2000; Dwoskin et al., 2000; Dwoskin and Crooks, 2002).

Over the past decade, intensive efforts have been made to identify the genes encoding the enzymes involved in the biosynthesis of isoquinoline, indole, tropane, and purine alkaloids (Croteau et al., 2000; Facchini et al., 2004). However, the molecular mechanisms underlying the biosynthesis of Lys-derived alkaloids are poorly understood. In this article, we discuss recent advances in our understanding of the Lys-derived alkaloids, especially the genes involved in the quinolizidine alkaloid (QA) biosynthetic pathway. We highlight the novel finding of Lys decarboxylase (LDC) from plants and discuss how it evolved from primary metabolism to alkaloid biosynthesis. Finally, we consider how to apply current advanced technologies to the study of unidentified alkaloid biosynthetic pathways.

## BIOSYNTHESIS OF LYS-DERIVED ALKALOIDS

L-Lys is the homologue of L-Orn, a precursor of pyrrolidine, tropane, and pyrrolizidine alkaloids. The extra methylene group in Lys participates in forming 6-membered piperidine rings, while Orn participates in forming 5-membered pyrrolizidine rings (Dewick, 2001; Poupon et al., 2011). Biosynthetic pathways of several potential Lys-derived alkaloids have been proposed based on tracer experiments with labeled precursors (Leistner and Spenser, 1973 and references therein). Feeding studies demonstrated that the first step in piperidine, quinolizidine, and lycopodium alkaloid biosynthesis is the decarboxylation of L-Lys into cadaverine by LDC (EC 4.1.1.18). Oxidative deamination of cadaverine by copper amine oxidase (CuAO, EC 1.4.3.22) yields 5-aminopentanal, which is spontaneously cyclized to Δ^1^-piperideine Schiff base (Leistner and Spenser, 1973; Gupta et al., 1979; Golebiewski and Spenser, 1988; Saito and Murakoshi, 1995; Ma and Gang, 2004). In contrast, indolizidine alkaloids are formed from L-Lys via L-pipecolic acid which is formed via the aldehyde and Schiff base, with retention of the nitrogen of the α-amino acid group (**Figure 1**; Harris et al., 1988a,b; Wickwire et al., 1990; Dewick, 2001). Δ^1^-Piperideine and L-pipecolic acid are universal intermediates that can be modified by condensation, hydrolysis, or methylation to produce diverse Lys-derived alkaloids (**Figure 1**; Evans, 2009).

The genes and enzymes involved in the biosynthesis of Lys-derived alkaloids are not well documented. The best-studied pathway at the genetic level is the one leading to the formation of QAs, which will be discussed in the following section. One other study addresses partially purified piperoyl-CoA: piperidine *N*-piperoyltransferase (EC 2.3.1.145) from* P. nigrum *which catalyzes the synthesis of piperine in the presence of piperoyl-CoA and piperidine (Geisler and Gross, 1990).

## QUINOLIZIDINE ALKALOIDS

Quinolizidine alkaloids are a group of alkaloids possessing a quinolizidine ring or a piperidine ring (Saito and Murakoshi, 1995). QAs occur mainly in the family Leguminosae, especially in the genera *Lupinus*, *Baptisia*, *Thermopsis*, *Genista*, *Cytisus*, and *Sophora *(Ohmiya et al., 1995). More than 500 *Lupinus* species have been described and 200–300 species are distributed in North and South America (Wink et al., 1995). QAs are important as potential sources of medicine. They have a broad range of pharmacological properties, including cytotoxic, oxytocic, antipyretic, antibacterial, antiviral, and hypoglycemic activities, as determined by *in vivo* pharmacological screening (Saito and Murakoshi, 1995). Some QA-containing plants, for example *Sophora flavescens,* have been used as sources of crude drugs in Chinese–Japanese medicine (KAMPO; Tang and Eisenbrand, 1992).

**FIGURE 2:**
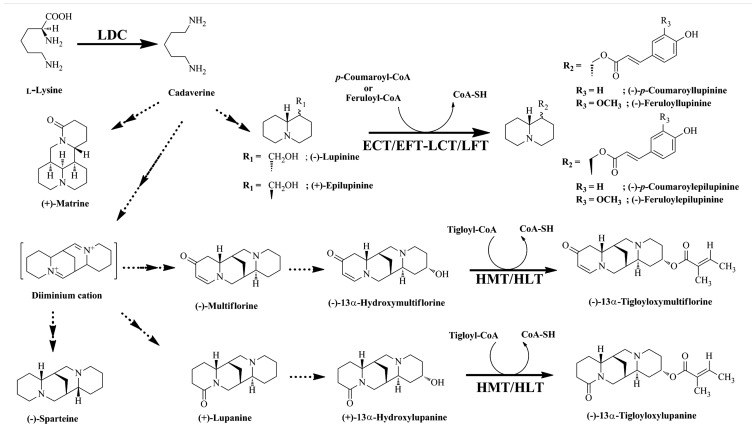
** Biosynthetic pathway of quinolizidine alkaloids**. Quinolizidine alkaloids are synthesized by the decarboxylation of Lys, yielding cadaverine, which is further modified by various reactions as shown. The metabolites derived from the pathway are shown. Enzymes involved in the synthesis of these alkaloids are indicated: LDC, Lys decarboxylase; HMT/HLT, tigloyl-CoA: (–)-13α-hydroxymultiflorine/(+)-13α-hydroxylupanine*O*-tigloyltransferase; ECT/EFT-LCT/LFT, *p*-coumaroyl-CoA/feruloyl-CoA: (+)-epilupinine/(–)-lupinine *O*-coumaroyl/feruloyltransferase.

Quinolizidine alkaloids are synthesized through the cyclization of the cadaverine unit (Golebiewski and Spenser, 1988), which is produced through the action of LDC (**Figure 2**; Saito and Murakoshi, 1995). Various QA skeletons are further modified by tailoring reactions (e.g., dehydrogenation, oxygenation, or esterification) to yield several hundred structurally related alkaloids (Ohmiya et al., 1995). *Lupinus* accumulates two types of QA esters, the derivatives of lupinine and lupanine. QA esters are assumed to be end products of biosynthesis and storage forms (Ohmiya et al., 1995). Two acyltransferases (ATs) involved in QA ester biosynthesis have been identified and characterized: (–)-13α-hydroxymultiflorine/(+)-13α-hydroxylupanine *O*-tigloyltransferase (HMT/HLT; EC 2.3.1.93), which catalyzes the transfer of the tigloyl group from tigloyl-CoA to (–)-13α-hydroxymultiflorine or (+)-13α-hydroxylupanine, and *p*-coumaroyl-CoA/feruloyl-CoA: (+)-epilupinine/(–)-lupinine *O*-coumaroyl/feruloyltransferase (ECT/EFT-LCT/LFT), which catalyzes the transfer of *p*-coumaroyl-CoA or feruloyl-CoA to the hydroxyl moiety of (+)-epilupinine or (–)-lupinine (**Figure 2**; Saito et al., 1992, 1993; Suzuki et al., 1994). However, only HMT/HLT has been cloned and characterized at the molecular level (Okada et al., 2005). HMT/HLT belongs to the BAHD super family, a plant acyl-CoA-dependent AT gene family that transfers acyl groups from their CoA esters to various plant metabolites (D’Auria, 2006).

An investigation of an alkaloid-rich “bitter” cultivar and an alkaloid-poor “sweet” cultivar of *L. angustifolius* using random amplified polymorphic DNA (RAPD) showed that total DNA polymorphisms did not differ substantially between the two cultivars (Hirai et al., 2000). A cDNA-amplified fragment length polymorphism (cDNA-AFLP) was then used to isolate the genes specifically expressed in the bitter plant; however, no bitter-specific gene was isolated (Hirai et al., 2000). The PCR-select subtraction technique was then used to profile differentially expressed genes in the two cultivars. Among 71 bitter-specific fragments, three genes exhibited homologies to Orn decarboxylase (ODC, EC 4.1.1.17), CuAO, and AT, representing potential candidates for structural genes encoding QA biosynthetic enzymes (Bunsupa et al., 2011). Further study showed that the AT might be involved either in the formation of QA esters or* N*-acylated polyamine conjugates (Bunsupa et al., 2011) and ODC was identified as Lys/Orn decarboxylase (L/ODC; see below) (Bunsupa et al., 2012). In addition, partial sequences encoding candidate transcription factors that might regulate the expression of genes in QA biosynthesis were obtained; these included DNA binding protein EREBP-3, leucine zipper protein, and coronatine-insensitive 1, which provide clues about the regulation of the QA biosynthetic pathway (Bunsupa et al., 2011). Genomic PCR of *L*. *angustifolius* AT and LDC indicated the presence of at least one copy of both genes in the genomes of both the bitter and sweet cultivars. However, transcripts of both genes were expressed only in the bitter cultivar. This finding is interesting in terms of the regulation of the entire QA biosynthetic pathway (Bunsupa et al., 2011, 2012).

## A NOVEL LYS DECARBOXYLASE FROM QUINOLIZIDINE ALKALOIDS PRODUCING PLANTS

Lys decarboxylase and ODC are key enzymes involved in the formation of cadaverine and putrescine by the decarboxylation of Lys and Orn, respectively. Cadaverine, putrescine, and other aliphatic polyamines, such as spermidine and spermine, are involved in a number of growth and developmental processes (Bagni and Tassoni, 2001). Unlike plant ODCs, plant LDCs have not been well studied at the molecular level. There are several studies on crude protein extracts or partially purified protein extracts from *Glycine max *(Kim et al., 1998; Ohe et al., 2009) and *L. polyphyllus *(Hartmann et al., 1980). There was speculation that a member of group IV of the PLP-dependent amino acid decarboxylases might be responsible for the production of cadaverine from Lys (Sandmeier et al., 1994) and that ODC may also have LDC activity in plants that make cadaverine-derived alkaloids (Sinclair et al., 2004).

Recently, we isolated a cDNA of L/ODC from *L*. *angustifolius* (*La-L/ODC*) using differential transcript screening in QA-producing and non-producing cultivars (Bunsupa et al., 2011, 2012). We also obtained *L/ODCs *cDNAs from *S*. *flavescens* and *Echinosophora koreensis*, which also produce QAs. Recombinant L/ODCs from QA-producing plants almost equally catalyzed the decarboxylation of L-Lys and L-Orn unlike the known ODCs that only accept Orn (Boeker and Fischer, 1983; Bunsupa et al., 2012). *In vivo* experiments of *La-L/ODC *expression in *Arabidopsis thaliana* and *Nicotiana tabacum* provided evidence that this enzyme is involved in the first step of QA biosynthesis (Bunsupa et al., 2012). This is the first plant LDC identified at the molecular level by cDNA cloning and the second QA biosynthesis enzyme for which the cDNA has been identified.

Identification of La-L/ODC answers a longstanding question about the molecular entity of LDC activity in plants. We found LDCs in plant species that produce QAs. A point mutation from His-344 to Phe-344 (La-L/ODC numbering) is key for La-L/ODC to exhibit substrate promiscuity for Lys and Orn. This amino acid substitution has presumably occurred via adaptive evolution of LDC within the QA-producing plants to expand substrate accessibility to Lys (Bunsupa et al., 2012). These results reveal how plants have been opportunistic in adapting primary metabolic processes to permit the diversification of secondary metabolism, in this case, substrate promiscuity. Accordingly, the mutation in this position may be found not only in QA-producing Leguminosae but also in plants that produce cadaverine-derived alkaloids. This finding is a breakthrough in the understanding of both QA biosynthesis and LDC occurrence and function in plants.

A major safety issue of lupine-based foods is the presence of QAs, which are toxic to human and mammals. Genetic mapping of *L*. *angustifolius* and *L. albus *has been completed, which enables the mapping of the genes controlling key domestication traits, including alkaloid production, water permeability, flowering time, pod shattering, pigment production in seeds, and anthracnose resistance (Boersma et al., 2005; Nelson et al., 2006; Phan et al., 2006). This information will be useful not only to the *Lupinus *research community but also to other legume research communities studying molecular breeding and evolution in leguminous plants. The identified La-L/ODC provides a good candidate enzyme as an entry-point enzyme of QA biosynthesis for metabolic engineering to manipulate the production of QAs.

## COMPARTMENTALIZATION AND SUBCELLULAR LOCALIZATION

The subcellular compartmentalization of alkaloid biosynthetic enzymes is as complex as the cell type-specific localization of gene transcripts, enzymes, and metabolites (Ziegler and Facchini, 2008). The majority of QAs are synthesized in shoot tissues. Of the QAs that accumulate in seeds, half are synthesized *in situ* and half are translocated primarily by phloem (Lee et al., 2007). The concentration of QAs in seeds decreases during germination and development and increases as soon as new leaves are formed (Wink and Witte, 1985). The biosynthesis of QAs occurs mainly in the green parts of plants where the enzymes for Lys, cadaverine, and lupanine synthesis are present (Wink and Hartmann, 1979, 1982; Wink and Mende, 1987). La-L/ODC is localized in chloroplasts, while HMT/HLT localized in mitochondria. ECT is assumed to be present in another organelle, but not in mitochondria (Suzuki et al., 1996; Bunsupa et al., 2012).

## ENGINEERING IN QA-PRODUCING PLANTS

The concentration of QAs in cell suspension cultures of *Lupinus* and related plants is approximately 100–1000 times lower than that of concentration in leaves of differentiated plants (Hartmann et al., 1980; Hernandez et al., 2011). In cell suspension cultures, the storage tissue is probably repressed; thus, the QAs cannot be stored in large quantities and are degraded rapidly either inside the cells or in the cell culture medium (Wink, 1987). In callus, the concentration of QAs is correlated to the amount of chlorophyll in the cells (Saito et al., 1989a,b).

*Lupinus *spp. are the major grain legumes in Australia and have been used as feed supplements. In the past decade, they have gained attention as ingredients for human food because of their high percentage of protein (34–43% of dry matter) and acceptable content of essential amino acids (Sujak et al., 2006). To genetically engineer plants successfully, a routine transformation system is needed. Thus, it is necessary to develop a transformation system for *Lupinus* lines to confer desired traits, such as herbicide resistance, resistance to certain pathogens, increased pod and seed set, and improved seed quality (Pigeaire et al., 1997). Many transformation systems for *Lupinus* sp. have been developed; however, only a few have been successful. For example, shoot regeneration using *Agrobacterium*-mediated gene transfer to preorganized meristematic tissue combined with axillary regeneration has been successful in *L*. *angustifolius *(Pigeaire et al., 1997), *L. luteus *(Li et al., 2000), and *L. mutabilis *(Babaoglu et al., 2000). However, transformation efficiency is poor and transformation in some *Lupinus* species and even in different cultivars of the same species is difficult. In *L*. *mutabilis*, the transformed roots synthesize isoflavones, but not QAs (Babaoglu et al., 2004). Genetic engineering of *L. angustifolius* by over expressed *sun flower seed albumin* gene has succeeded to enhance methionine levels and increase nutritive value of seeds of transgenic *Lupinus* plants (Molvig et al., 1997).

## FUTURE PROSPECTS

In the past few years, progress has been made in the elucidation of alkaloid biosynthetic pathways and their regulation. However, only two genes, HMT/HLT and L/ODC, have been isolated and characterized so far. Rapid progress in the development of integrative approaches, such as genomics, transcriptomics, proteomics, and metabolomics, has provided essential information for the understanding of many complex biological processes at different levels (Fukushima et al., 2009; Yonekura-Sakakibara and Saito, 2009). Comparative and integrative analyses of such “omics” data will facilitate the determination of gene function and pinpoint rate limiting steps and factors in the biosynthetic pathway of secondary metabolites. Transcriptomic and metabolomic approaches, particularly transcriptome coexpression network analysis, have been used to identify genes involved in specific pathways not only in *Arabidopsis *but also in crops and medicinal plants. Such strategies have proven to be useful in fundamental plant biology and applied biotechnology (Ziegler et al., 2006; Hirai et al., 2007; Saito et al., 2008: Seki et al., 2008; Yonekura-Sakakibara et al., 2008, 2012; Liscombe et al., 2009; Okazaki et al., 2009; Saito and Matsuda, 2010; Okazaki and Saito, 2012).

Advances in sequencing technology, next-generation sequencing, and computer technology for processing of large data sets are opening a new era in plant science. It is estimated that the cost of DNA sequencing drops at a faster rate than the cost of computer data processing (Ehrhardt and Frommer, 2012). Therefore, the development of sequencing techniques with high-throughput will likely be applied to alkaloid-producing plants and provide the basis for many sequence-based approaches that are now limited to model plants such as *Arabidopsis* and rice (Ziegler and Facchini, 2008). Today, many researchers are making progress in plant genome and transcriptome sequencing. For instance, the 1KP project aims to generate large-scale gene sequence information for 1000 different plants, including a wide variety of species from algae to land and aquatic plants, to understand the vast biodiversity that has barely been touched by genomics to date^1^. In addition, the medicinal plant consortium project focuses on providing transcriptomic and metabolic resources for 14 key medicinal plants, for instance *Atropa belladonna* and *Camptotheca acuminata*, to the worldwide research community for the advancement of drug production and development^2^. Recently, we started the deep transcriptome sequencing of Japanese medicinal plants and both cultivars of *L. angustifolius* to obtain more information about the enzymes and transcription factors involved in the production of plant secondary metabolites. Such publicly accessible databases will provide novel information for the research community and ultimately accelerate the progress of the study of plant science.

The predicted function or *in vitro* characterization of alkaloid biosynthetic enzymes and other components must be confirmed *in vivo* (Ziegler and Facchini, 2008). However, there is no reliable and highly efficient transformation protocol for *Lupinus* and related plants. Virus induced gene silencing (VIGS), a technology that exploits an RNA-mediated antiviral defense mechanism, is a fast method for the generation of transient transformed plants (Lu et al., 2003). VIGS has been used widely in plants for analysis of gene function and has been adapted for high-throughput functional genomics (Burch-Smith et al., 2004). Thus, VIGS might be a powerful tool for assessing and characterizing the function of candidate genes in the QA biosynthetic pathway. Further investigations on inter- and intracellular transport of pathway intermediates and products and on cellular and subcellular localization of enzymes are also important because they are crucial factors in controlling the accumulation of target metabolites in plants (Facchini et al., 2004).

## CONCLUSION

Knowledge of the biosynthetic pathway of Lys-derived alkaloids is limited. The best-studied pathway at the genetic level is QA biosynthesis; however, only two genes have been identified. Application of current technologies, such as next-generation sequencing, high-end proteomics, and metabolomics approaches, is needed to study secondary metabolic pathways for a better understanding of metabolic networks. Such information will aid in the development of successful strategies in genetic engineering of secondary metabolites in plants.

## Conflict of Interest Statement

The authors declare that the research was conducted in the absence of any commercial or financial relationships that could be construed as a potential conflict of interest.

## Footnotes

1 http://www.onekp.com/project.html

2 http://medicinalplantgenomics.msu.edu/index.shtml
